# ZIKV infection activates the IRE1-XBP1 and ATF6 pathways of unfolded protein response in neural cells

**DOI:** 10.1186/s12974-018-1311-5

**Published:** 2018-09-21

**Authors:** Zhongyuan Tan, Wanpo Zhang, Jianhong Sun, Zuquan Fu, Xianliang Ke, Caishang Zheng, Yuan Zhang, Penghui Li, Yan Liu, Qinxue Hu, Hanzhong Wang, Zhenhua Zheng

**Affiliations:** 1 CAS Key Laboratory of Special Pathogens and Biosafety, Center for Emerging Infectious Diseases, Wuhan Institute of Virology, Chinese Academy of Sciences, Wuhan, 430071 China; 2 University of Chinese Academy of Sciences, Beijing, 100049 China; 3 College of Veterinary Medicine, Huazhong Agricultural University, Wuhan, 430070 China; 4 State Key Laboratory of Virology, Wuhan Institute of Virology, Chinese Academy of Sciences, Wuhan, 430071 China

**Keywords:** Zika virus, Neural cell, Neuropathogenesis, ER stress, Unfolded protein response

## Abstract

**Background:**

Many viruses depend on the extensive membranous network of the endoplasmic reticulum (ER) for their translation, replication, and packaging. Certain membrane modifications of the ER can be a trigger for ER stress, as well as the accumulation of viral protein in the ER by viral infection. Then, unfolded protein response (UPR) is activated to alleviate the stress. Zika virus (ZIKV) is a mosquito-borne flavivirus and its infection causes microcephaly in newborns and serious neurological complications in adults. Here, we investigated ER stress and the regulating model of UPR in ZIKV-infected neural cells in vitro and in vivo.

**Methods:**

Mice deficient in type I and II IFN receptors were infected with ZIKV via intraperitoneal injection and the nervous tissues of the mice were assayed at 5 days post-infection. The expression of phospho-IRE1, XBP1, and ATF6 which were the key markers of ER stress were analyzed by immunohistochemistry assay in vivo. Additionally**,** the nuclear localization of XBP1s and ATF6n were analyzed by immunohistofluorescence. Furthermore, two representative neural cells, neuroblastoma cell line (SK-N-SH) and astrocytoma cell line (CCF-STTG1), were selected to verify the ER stress in vitro. The expression of BIP, phospho-elF2α, phospho-IRE1, and ATF6 were analyzed through western blot and the nuclear localization of XBP1s was performed by confocal immunofluorescence microscopy. RT-qPCR was also used to quantify the mRNA level of the UPR downstream genes in vitro and in vivo.

**Results:**

ZIKV infection significantly upregulated the expression of ER stress markers in vitro and in vivo. Phospho-IRE1 and XBP1 expression significantly increased in the cerebellum and mesocephalon, while ATF6 expression significantly increased in the mesocephalon. ATF6n and XBP1s were translocated into the cell nucleus. The levels of BIP, ATF6, phospho-elf2α, and spliced *xbp1* also significantly increased in vitro. Furthermore, the downstream genes of UPR were detected to investigate the regulating model of the UPR during ZIKV infection in vitro and in vivo. The transcriptional levels of *atf4*, *gadd34*, *chop*, and *edem-1* in vivo and that of *gadd34* and *chop* in vitro significantly increased.

**Conclusion:**

Findings in this study demonstrated that ZIKV infection activates ER stress in neural cells. The results offer clues to further study the mechanism of neuropathogenesis caused by ZIKV infection.

**Electronic supplementary material:**

The online version of this article (10.1186/s12974-018-1311-5) contains supplementary material, which is available to authorized users.

## Background

The mosquito-borne flavivirus, Zika virus (ZIKV), was first isolated in 1947 from a rhesus monkey in the East African country of Uganda [1]. ZIKV infection in humans was first reported in 1952 and only 14 cases of sporadic infection have been previously documented until an outbreak was reported by physicians on Yap Island in 2007 [2–4]. The unprecedented epidemics of ZIKV among the Americas in 2015 have raised alarm due to its rapid transmission and association with microcephaly in newborns and serious neurological complications in adults, such as Guillain–Barre syndrome [4–7]. The World Health Organization declared a public health emergency to maintain international concern regarding the virus [8]. The relationship between ZIKV infection and neurodevelopment abnormalities has attracted more and more attention [5, 7, 9, 10]. ZIKV, like other members of the *Flavivirus* genus, is a positive (+) single-strand RNA virus. An approximately 10.7 kb genome of ZIKV encodes a single polyprotein precursor that is posttranslationally cleaved into three structural proteins (C, prM/M, and E) and seven nonstructural proteins (NS1, NS2A, NS2B, NS3, NS4A, NS4B, and NS5) by viral and host proteases [11–13]. Nonstructural proteins induce the formation of a membranous network with ER where viral replication occurs [14]. Immature virions assemble within the ER, where viral RNA is complexed with the C protein and packaged into an ER-derived lipid bilayer containing heterodimers of prM and E proteins [15]. Immature virions then bud into the ER lumen and are transported through the *trans*-Golgi network. In the Golgi apparatus, glycan modification and structural cleavage (furin-mediated cleavage of the prM to M) are accomplished, and mature infectious virions are secreted into extracellular space via exocytosis [14, 15].

The ER is an important location for posttranslational modification, folding, and oligomerization of secretory and cell surface proteins. It is also a main intercellular signal-transducing organelle for responding to environmental changes. Many viruses depend on the extensive membranous network of the ER for their translation, replication, and packaging [16]. The *Flaviviridae* family, including the dengue virus (DENV) [16, 17], West Nile virus (WNV) [18, 19], yellow fever virus (YFV), hepatitis C virus (HCV) [20], and Japanese encephalitis virus (JEV) [17], depend on the ER for their life cycles and are called endoplasmic reticulum tropic (ER-tropic) viruses [16]. Infection by an ER-tropic virus disrupts the normal ER function, and then ER stress is induced. To alleviate ER stress, the UPR is activated and mainly functions in translational attenuation, protein folding, protein degradation, and cellular apoptosis [21, 22]. PKR-like ER kinase (PERK), transcription factor 6 (ATF6) and inositol-requiring enzyme 1 (IRE1) are the sensors of the UPR pathway. In unstressed cells, the ER chaperone immunoglobulin heavy-chain-binding protein (BIP) binds to the ER luminal domain of the three sensors. Under the condition of ER stress, however, BIP is dissociated from the three sensors and preferentially binds to misfolded and unfolded proteins. Then, the three response pathways are activated to deal with different ER stress states in a time-dependent manner [16]. Phosphorylated PERK phosphorylates the α-subunit of eukaryotic translation initiation factor 2 (eIF2α). Phosphorylated eIF2α (phospho-eIF2α) forms a complex with guanine nucleotide exchange factor (eIF2B) and inhibits catalysis of GDP-GTP exchange, thereby leading to the translation attenuation and contradictory expression by activating transcription factor 4 (ATF4). Activated ATF4 upregulates a series of genes related to encoding metabolism and redox regulation and helps cells recover from ER stress. Growth arrest and DNA damage-inducible protein (GADD34), which is regulated by ATF4, interacts with protein phosphatase 1 (PP1) to dephosphorylate eIF2α, thereby acting as a negative feedback loop to restore protein translation [23]. PERK-mediated eIF2α phosphorylation is triggered in early DENV-2 infection and suppressed in mid and late DENV-2 infection because the inhibition of eIF2α phosphorylation is necessary for viral protein synthesis [16]. The RNase activity of phosphorylated IRE1 (phospho-IRE1) has only one substrate, X-box binding protein 1 (XBP1), and removes a 26-nucleotide intron from unspliced *xbp1*mRNA (*xbp1u)* [24, 25]. The spliced *xbp1*mRNA (*xbp1s*) results in a frameshift mutation and encodes spliced XBP1 (XBP1s), a transcription factor that binds to the sequences of ER stress response elements (ERSEs) and UPR elements (UPREs), and upregulates the transcription of ER-association degradation (ERAD) proteins, proapoptotic proteins, and some ER chaperones [26]. JEV infection activates the IRE1-XBP1 pathway and has a beneficial effect on the activation of the regulated IRE1-dependent decay (RIDD) pathway during the viral life cycle [17, 27]. Cleaved ATF6 (ATF6n) also can bind to the ERSE and UPRE sequences and upregulate transcription chaperones, foldases, and lipid synthesis genes [26]. ATF6 is required for efficient WNV replication by maintaining cell viability and modulating the innate immune responses [28]. In brief, ER is an important location in viral life cycle, and UPR is closely associated with the regulation of viral replication. ZIKV is a flavivirus that its life cycle also closely depends on the extensive membranous network of the ER [29]. However, whether ZIKV activates and benefits from ER stress to regulate viral replication needs to be investigated.

Many neurological diseases are linked to abnormal protein accumulation in the ER and activation of UPR to deal with ER stress [30]. Khajavi and Lupski reported that the UPR is responsible for the demyelination in peripheral neuropathy [31]. Yang and Paschen proposed that the UPR dysfunction after brain ischemia contributes to neuronal death [32]. Several studies found a clear activation of the UPR in toxicological models of Parkinson’s disease. ATF6, XBP1, and pro-apoptotic transcription factor CCAT/enhancer-binding protein (CHOP) play functional roles in controlling dopaminergic neuron survival [33]. In addition, whether the UPR plays an important role in the neuropathogenesis caused by ZIKV infection has yet to be studied in animal models or cellular level. In the present study, we found that ZIKV infection activated ER stress both in vitro and in vivo, showing that the expression of ER stress markers, namely, BIP, cleaved ATF6, phospho-IRE1, and phospho-eIF2α, significantly increased. The regulating model of UPR was developed, with ZIKV infection activating the IRE1-XBP1 pathway to regulate cellular apoptosis mediated by CHOP. This study could serve as a reference for elucidating ZIKV neuropathogenesis.

## Methods

### Cells, viruses, and infection

*Aedes albopictus* clone C6/36 cells (ATCC-CRL-1660) were cultured in Dulbecco’s Modified Eagle’s medium. SK-N-SH cells (ATCC-HTB-11) and African green monkey kidney epithelial Vero cells (CCL-81, American Type Culture Collection) were cultured in MEM. CCF-STTG1 cells (ATCC-CRL-1718) were cultured in RPMI 1640. All cells were maintained in a medium containing 10% fetal bovine serum (Life Technology, Australia) in 5% CO_2_ at 37 °C. ZIKV strain (Zika virus/SZ01/2016/China, GenBank: KU866423.2) was obtained from the Wuhan Institute of Virology, Chinese Academy of Science [34]. It was propagated using C6/36 cells. Virus titers were measured using 50% tissue culture infectious dose (TCID50) in Vero cells and analyzed using Reed–Muench formula [35]. The cells were infected with ZIKV at a multiplicity of infection (MOI) of 5. Positive controls were treated with tunicamycin at a final concentration of 2 μM.

### Animals and treatments

C57BL/6 mice deficient in type I and II IFN receptors (AG6 mouse, *ifnagr*^−/−^) were acquired from Prof. Qibin Leng in the Institute Pasteur of Shanghai, Chinese Academy of Sciences. Three-week-old AG6 mice were infected with 1 × 10^5^ PFU/mouse (each dose, *N* = 3) via intraperitoneal injection. Mock-infected controls were administered with PBS through the same route. At 5 days post infection (dpi), the mice were perfused with 4% paraformaldehyde, and the brain tissues were obtained and treated according to further assay.

### Western blot analysis

Cells were washed once with PBS and then lysed on ice in RIPA buffer (Beyotime) supplemented with phenylmethanesulfonyl fluoride (PMSF) (Beyotime) and protease inhibitor cocktail tablets (Roche) in accordance with the manufacturer’s instructions. Total protein was separated via SDS polyacrylamide gel electrophoresis containing 10% polyacrylamide gels and then transferred onto Immobilon-P polyvinylidene fluoride membranes (Millipore) in transfer buffer [30 mM Tris, 200 mM glycine, 20%(*V*/*V*) methanol] for 150 min at 4 °C. Immunoblots were blocked using 5% bovine serum albumin (BSA) dissolved in TBS-T for 1 h at 37 °C and then incubated with primary antibodies diluted using primary antibody dilution buffer (Beyotime) overnight. After washing with TBS-T three times for 7 min each, immunoblots were incubated with horseradish peroxidase (HRP)-conjugated secondary antibodies diluted using TBS-T with 0.5% BSA for 1 h at 37 °C. After washing with TBS-T five times for 7 min each, immunoblots were visualized and analyzed by using a Bio-Rad imaging system with an Immobilon Eastern Chemiluminescent HRP substrate (Millipore). The band was analyzed by using Image Lab 4.0.1.

The primary antibodies used in this study were as follows: ATF6 (Beyotime, 24,169–1-AP), phospho-IRE1 (Abcam, ab48187), BIP (Beyotime, AB310), phospho-elF2α (Cell Signaling Technology, #9721), total elf2α (Cell Signaling Technology, #9722), XBP1 (Abcam, ab37152), β-actin (Beyotime, AF0003), and ZIKV envelope protein (BioFront Technologies, BF-1176-56). All antibodies were diluted in accordance with the manufacturer’s protocol.

### RT-qPCR analysis

Total RNA was extracted from cells and mouse brains by using TRIZOL Reagent (Invitrogen) in accordance with the manufacturer’s protocol. The first strand cDNA was reverse transcribed from 1 μg of total RNA by using PrimeScript RT reagent Kit with gDNA Eraser (Takara) in accordance with the manufacturer’s protocol. Each 20 μL of quantitative PCR reaction mix contains cDNA, forward primer, reverse primer, and iTap™ Universal SYBR Green^®^ Supermix (Bio-Rad). Amplification in a Bio-Rad CFX real-time quantitative PCR system involved activation at 95 °C for 3 min followed by 40 amplification cycles of 95 °C for 5 s and 60 °C for 45 s. All primers for PCR are shown in Table 1. Virus RNA copies were quantified by the standard curve method and relative expression level of genes were analyzed by using the 2^−∆∆Ct^ method.

**Table 1:** Primers used for RT-qPCR assay

Gene	Forward primer (5′→3′)	Reverse primer (5′→3′)
ZIKV	AARTACACATACCARAACAAAGTG	TCCRCTCCCYCTYTGGTCTTG
*gadd34*-human	GATGGCATGTATGGTGAGCG	GAGACAAGGCAGAAGTAGAG
*atf4*-human	CACCGCAACATGACCGAAAT	GACTGACCAACCCATCCACA
*chop*-human	GAACCAGGAAACGGAAACAG	ATTCACCATTCGGTCAATCA
*xbp1s*-human	AAGAACACGCTTGGGAATGG	CTGCACCTGCTGCGGAC
T-*xbp1*-human	GACAGAGAGTCAAACTAACGTGG	GTCCAGCAGGCAAGAAGGT
*edem-1*-human	CTGGTGGAATTTGGGATTCT	GTATCATTGCTCCGGAGG
*gapdh*-human	GTCTCCTCTGACTTCAACAGCG	ACCACCCTGTTGCTGTAGCCAA
*bip*-mouse	CCATCCCGTGGCATAAAC	GACTCCTCCCACAGTTTCA
*ire1*	GGACAGGCTCAATCAAATGG	CGGTCAGGAGGTCAATAACA
*atf6*	TCGGTCAGTGGACTCTTATT	CCAGTGACAGGCTTATCTTC
*gadd34*-mouse	CCGCTTATCCCACATCAC	GGTTTGTATCCCGGAGCT
*atf4*-mouse	TTAGAGCTAGGCAGTGAAGTT	CTGTCATTGTCAGAGGGAGT
*chop*-mouse	CACATCCCAAAGCCCTCG	CGTTCTCCTGCTCCTTCTC
*xbp1s*-mouse	CTGAGTCCGCAGCAGGTG	GACCTCTGGGAGTTCCTCCA
T-*xbp1*-mouse	GGGACTACAGGACCAATAA	ACCATAGCCAGGAAACGT
*edem-1*-mouse	GGAGTCCCATTCTACAACC	GCAATCCGAAAGCCACCA
*gapdh*-mouse	AACGACCCCTTCATTGAC	TCCACGACATACTCAGCAC

### Confocal immunofluorescence microscopy

For localization assay, SK-N-SH and CCF-STTG1 cells were grown on glass slides with 5 × 10^4^ cell per dish. Mock, tunicamycin-treated, and ZIKV-infected cells were designed and manipulated as described above. At 24 h post infection, the slides were washed with PBS, fixed with 4% paraformaldehyde, and then permeabilized with 0.2% Triton X-100 for 15 min. The cells were blocked by using 5% BSA and 3% normal goat serum (NGS) dissolved in PBS for 1 h at 37 °C. The cells were then washed with 3% NGS dissolved in PBS two times and then incubated in primary antibodies (1:200) diluted with 3% NGS dissolved in PBS overnight at 4 °C. After washing five times, cells were incubated with Goat anti-Rabbit IgG secondary antibody, FITC (Thermo Fisher Scientific, #F-2765), and Goat anti-Mouse IgG secondary antibody, Texas Red®-X (Thermo Fisher Scientific, #T-6390) (1:200) diluted with 3% NGS dissolved in PBS for 1 h at 37 °C. After washing two times, cell nucleus was dyed with Hoechst 33258 for 10 min at 37 °C. Microscopic analysis was performed as previously described [36]. The primary antibody used in this study was as follows: XBP1 (Abcam, ab37152), ZIKV envelope protein (BioFront Technologies, BF-1176-56).

### Immunohistochemistry and immunohistofluorescence

For immunohistochemistry assay, the paraffin blocks of tissues were divided into 5-μm sections. After deparaffinization and rehydration, the sections were treated with 3% hydrogen dioxide solution for 30 min at room temperature and then washed two times with PBS for 5 min each. The sections were incubated in citrate buffer solution for 30 min at 95–100 °C, cooled, and then washed three times with PBS for 5 min. The sections were then blocked with 5% BSA for 1 h at room temperature and then incubated in primary antibodies (1:300) overnight at 4 °C. After washing three times, the sections were incubated in HRP-conjugated second antibodies for 1 h at 37 °C. Subsequently, the sections were covered with 0.02% DAB solution and counterstained with hematoxylin for 3 min. The sections were then washed for 10 min, dehydrated, transparentize, and then sealed with neutral resin sheets. Images were captured by using a NIS-Elements system under a Nikon 80iMicroscope. Aperio ImageScope viewing software was used to analyze the percentage of positivity (algorithm, positive pixel count). For immunohistofluorescence assay, the whole brain sections of AG6 mice were stained with DAPI, anti-ZIKV-ENV antibody, and anti-XBP1 or anti-ATF6 antibody. The experimental procedure accorded to the normal protocol. Images were captured by using a digital slice scanning analysis system (Pannoramic MIDI). The primary antibodies used in this study were as follows: XBP1 (Abcam, ab37152), ATF6 (Abcam, ab37149), phospho-IRE1 (Abcam, ab48187), ZIKV envelope protein (BioFront Technologies, BF-1176-56).

### Statistical analysis

All experiments were reproducible and carried out in triplicate. Data are represented as mean ± SD when indicated and Student’s *t* test was used for all statistical analyses with the GraphPad Prism 6.0 software. Differences were considered significant when *P* value was less than 0.05.

## Results

### ZIKV infection activates the IRE1-XBP1 and ATF6 pathways of UPR in the nervous tissues of the mouse brain

**Fig. 1:**
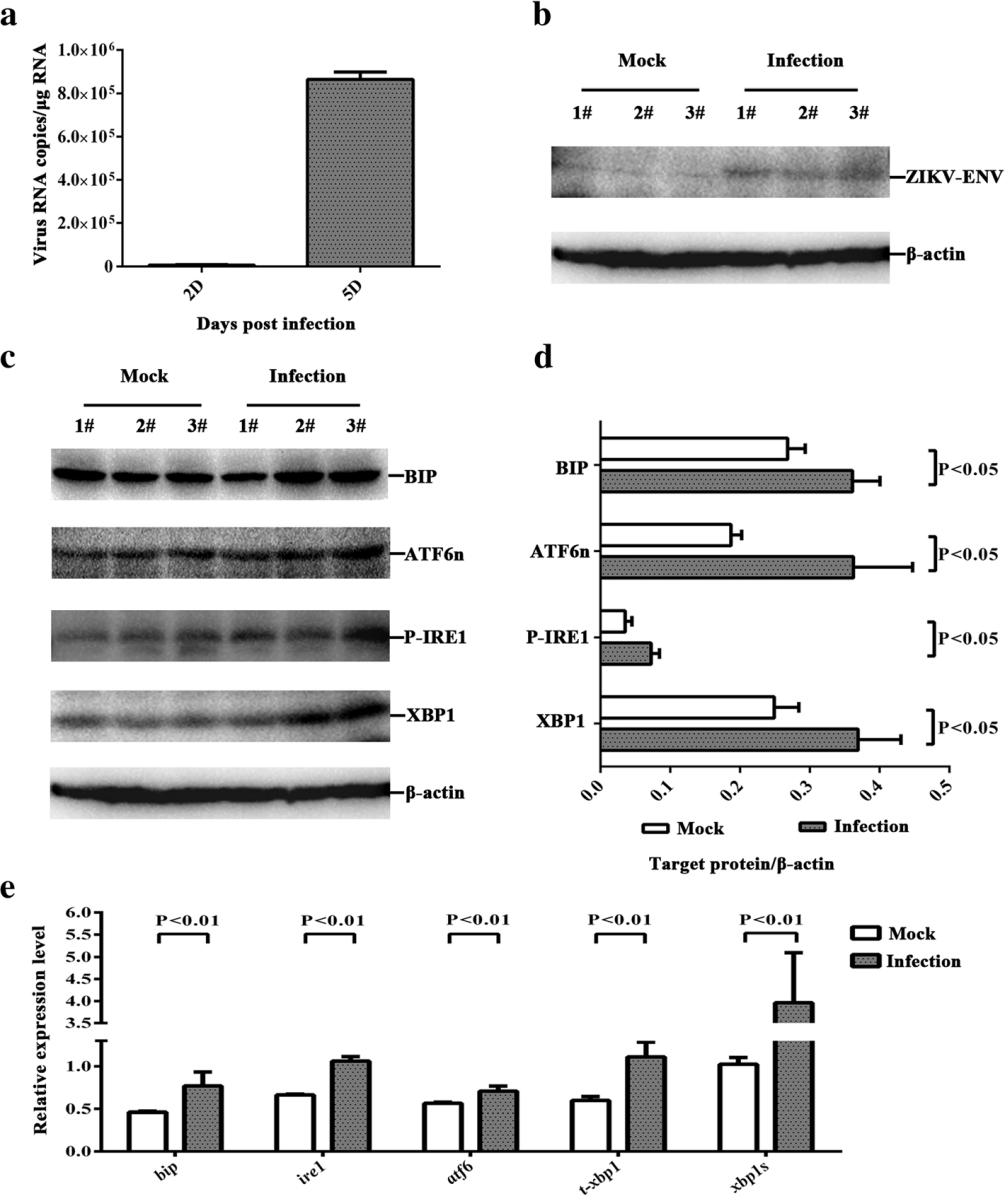
ZIKV infects the nervous tissues of the mouse brain and activates the ER stress markers**.** Three-week-old AG6 mice were infected with 1 × 10^5^ PFU/mouse (each dose, *N* = 3) via intraperitoneal injection. **a**. The total mRNA of the mouse brain was extracted, and ZIKV mRNA was detected at 2 dpi and 5 dpi via RT-qPCR. **b**, **c** The total lysates of mouse brain were analyzed at 5 dpi via western blot. An equal amount of lysates were analyzed with anti-ZIKV envelope protein antibody (**b**), anti-BIP antibody, anti-ATF6n antibody, anti-phoshpo-IRE1 antibody, and anti-XBP1 antibody (**c**). **d**. The band was analyzed by using Image Lab 4.0.1. The fold change of the *target protein*/*β-actin* was calculated. The protein expression of mock-infected and ZIKV-infected samples were normalized with the internal control β-actin. **e**. Primers that specifically amplified *bip*, *ire1*, *atf6*, *t-xbp1*, and *xbp1s* were used. The relative expression levels of the genes were calculated according to the 2^−∆∆*Ct*^ method. Data represented three independent experiments and *error bars* indicate mean ± SD. Statistical analyses were performed using multiple *t* tests (*N* = 3) (*P* < 0.05 or *P* < 0.01)

AG6 mice succumb to ZIKV infection and sustain high viral loads in many tissues, including the spleen, liver, kidney, serum, testes, brain, and spinal cord [37, 38]. To study whether ZIKV infection causes ER stress in the nervous tissues of the mouse brain, AG6 mice were infected with 1 × 10^5^ PFU of ZIKV (SZ01/2016/China). The total mRNA of the mouse brain was extracted for the detection of ZIKV mRNA by RT-qPCR, revealing 6.2 × 10^4^ copies/μg RNA at 2 dpi and 8.6 × 10^5^ copies/μg RNA at 5 dpi (Fig. 1a), respectively. Total lysates of mouse brain were analyzed via western blot and ZIKV envelope protein (ZIKV-ENV) could only be detected at 5 dpi (Fig. 1b). The cerebrum, cerebellum, and mesocephalon were extracted for immunohistochemistry assay, showing that these tissues were all infected by ZIKV (see Additional file 1: a, b). These results together indicate that ZIKV infects and replicates in the nervous tissues of the mouse brain, and this infection model can be used in future studies.

The ER chaperone BIP, which is dissociated from three UPR sensors under stress and acts as the primary indicator or regulatory factor [16], was analyzed by using total lysates and mRNA from the nervous tissues of the mouse brain. Following ZIKV infection, BIP expression significantly increased at the levels of protein and mRNA (Fig. 1c, d, e). These results suggest that BIP was likely activated to regulate the homeostasis of ER in the nervous tissues of the mouse brain infected with ZIKV.

IRE1-XBP1 is an important arm of the UPR. Phospho-IRE1 splices a 26-nt intron from full-length *xbp1u*, resulting in the formation of *xbp1s*. *Xbp1s* encodes the transcription factor XBP1s which activates the expression of genes involved in chaperone and protein degradation [39, 40]. Phospho-IRE1 and XBP1 were analyzed by western blot. Compared with mock-infected samples, the ZIKV-infected nervous tissues of the mouse brain exhibited an upregulation of phospho-IRE1 and XBP1 (Fig. 1c, d). The genes encoding IRE1 and XBP1 were also analyzed via RT-qPCR. As there are two forms of *xbp1*, primers that specifically amplified *xbp1s* and total *xbp1* (*t-xbp1*, including both *xbp1u* and *xbp1s*) were used [41]. We found that the expression of *ire1* increased. The ratio of *xbp1s*/t-*xbp1* between mock-infected and ZIKV-infected was 2.08-fold (Fig. 1e). Collectively, the findings demonstrated that ZIKV infection in the nervous tissues of the mouse brain activated phospho-IRE1 and induced the post-transcriptional cleavage of *xbp1*.

The targets of ATF6 are prominent ER-resident proteins involved in protein folding, such as BIP. Upon accumulation of the unfolded proteins, ATF6 is transported to the Golgi apparatus and cleaved by two proteases. The 50-kDa N-terminal cytosolic fragment, ATF6n, moves into the nucleus to activate the UPR genes [25]. The expression of ATF6 was analyzed by using total lysates and mRNAs from the nervous tissues of the mouse brain. We found that, following ZIKV infection, ATF6n expression was moderately upregulated at the protein level, and *atf6* at the mRNA level (Fig. 1c, d, e). The results indicated that ATF6 pathway was activated in the ZIKV-infected nervous tissues of the mouse brain.

**Fig. 2:**
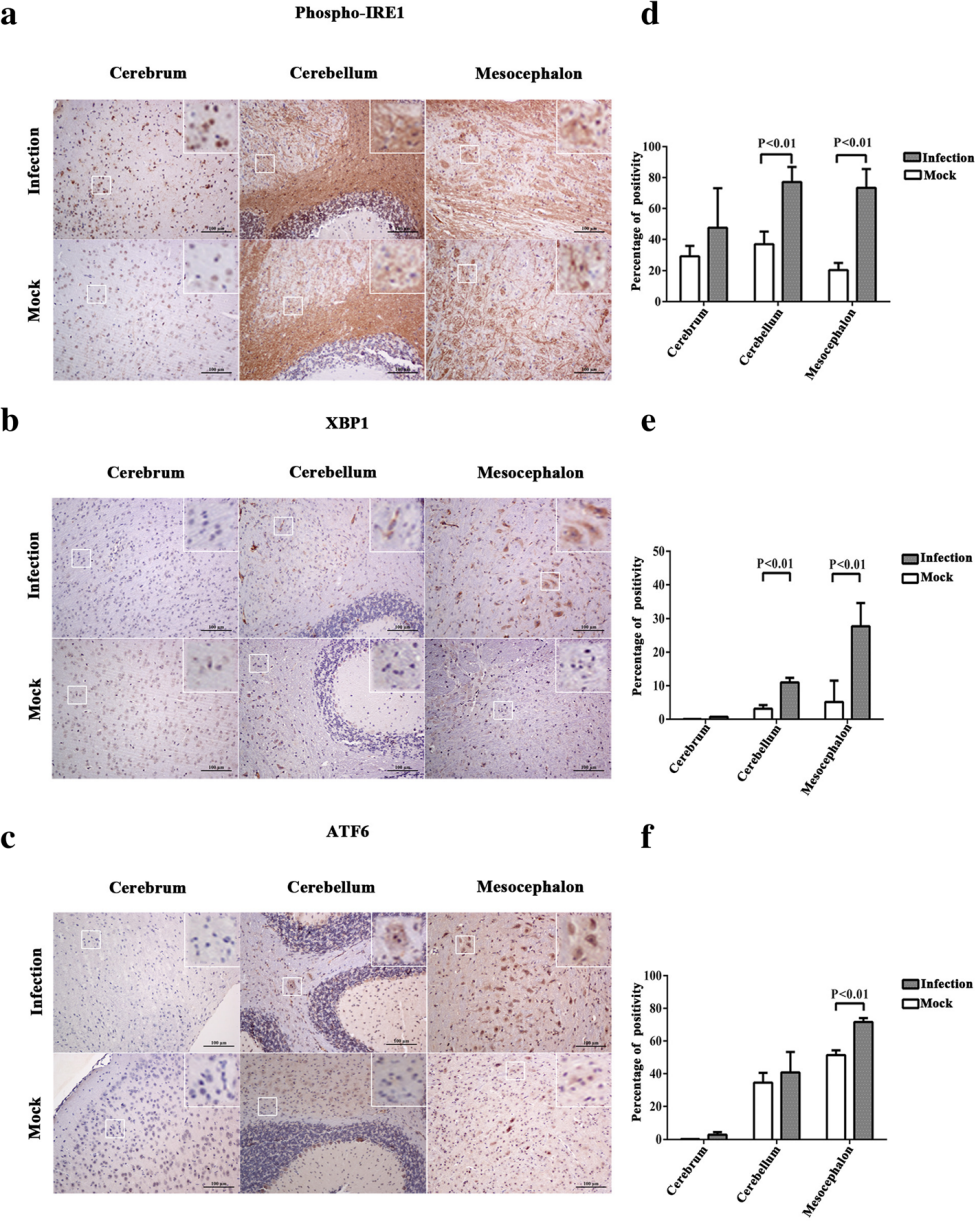
ZIKV infection upregulates the expression of phospho-IRE1, XBP1, and ATF6 in the mouse brain. Three-week-old AG6 mice were infected with 1 × 10^5^ PFU/mouse (each dose, *N* = 3) via intraperitoneal injection. **a**, **b**, **c** Sections of the cerebrum, cerebellum, and mesocephalon were obtained at 5 dpi, and anti-phospho-IRE1 antibody (**a**), anti-XBP1 antibody (recognizing both non-spliced and spliced isoforms of XBP1) (**b**), and anti-ATF6 antibody (recognizing both cleaved and full forms of ATF6) (**c**) were used for immunohistochemistry assay. Purple dots represent the cell nucleus. Brown spots represent the ZIKV envelope protein. A representative of three independent experiments is shown. Positive cells marked by white squares were magnified nine times and showed on the top-right corner. **d**, **e**, **f** Aperio ImageScope viewing software was used to analyze the percentage of positivity (algorithm, positive pixel count). Data represented three independent experiments and *error bars* indicate mean ± SD. Statistical analyses were performed using multiple *t* tests (*N* = 3) (*P* < 0.05 or *P* < 0.01)

**Fig. 3:**
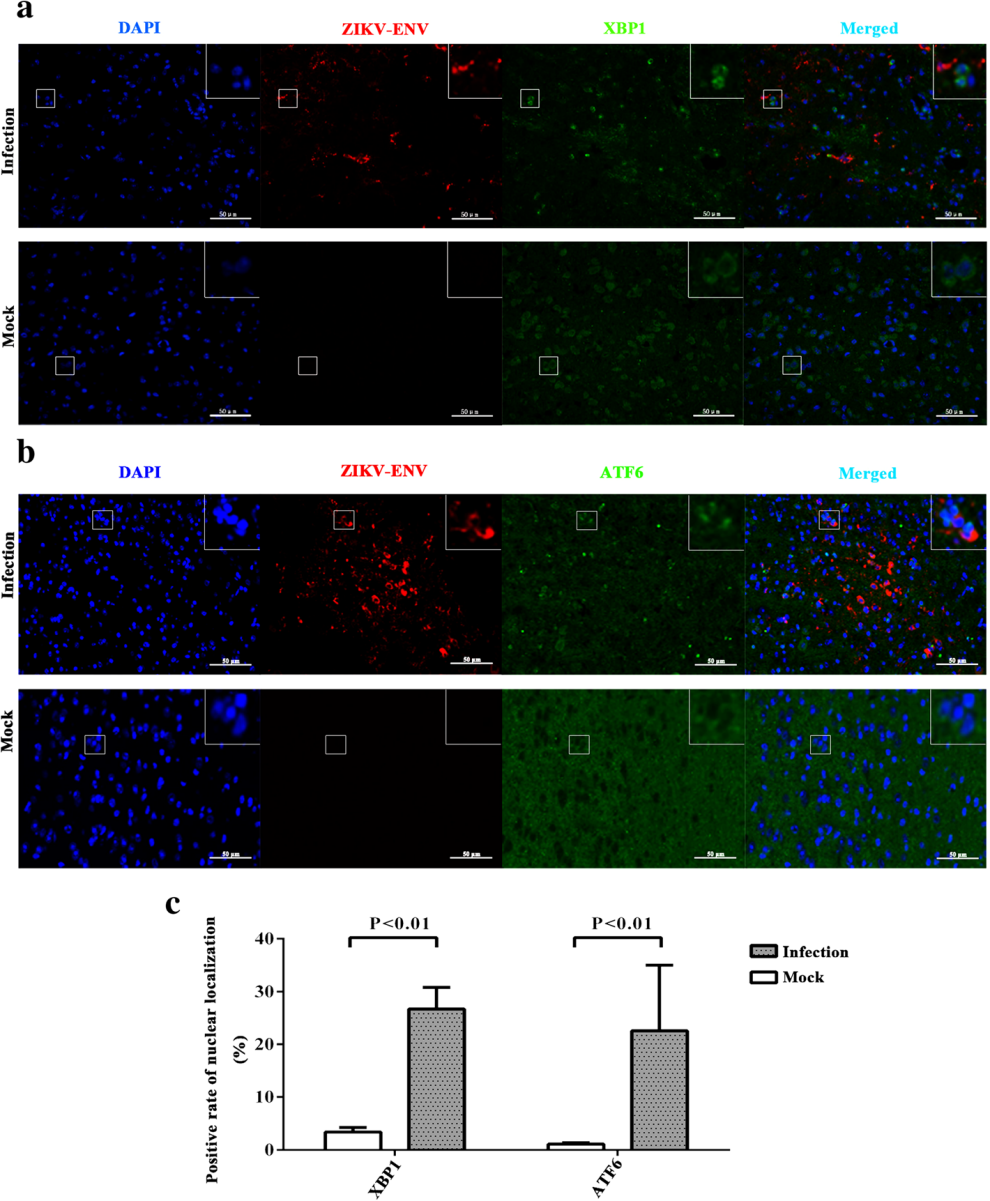
ZIKV infection stimulates ATF6n and XBP1s to translocate into the cell nuclei of neural cells in the mouse brain. Three-week-old AG6 mice were infected with 1 × 10^5^ PFU/mouse (each dose, *N* = 3) via intraperitoneal injection. **a**, **b** The whole brain sections of AG6 mice were stained with DAPI (blue), anti-ZIKV envelope protein antibody (red), and anti-XBP1 (**a**) or anti-ATF6 (**b**) antibody (green) and were used for immunohistofluorescence assay at 5 dpi. Images of the whole brain were captured by using a digital slice scanning analysis system (Pannoramic MIDI). A representative sub-area of mesocephalon is shown. Positive cells marked by white squares were magnified nine times and shown on the top-right corner. **c** Positive rates of nuclear localization were counted. Data represent three area of mesocephalon through random selection and *error bars* indicate mean ± SD. Statistical analyses were performed using multiple *t* tests (*N* = 3) (*P* < 0.05 or *P* < 0.01)

Sections from the cerebrum, cerebellum, and mesocephalon of AG6 mice were stained using anti-phospho-IRE1 antibody,anti-ATF6 antibody (recognizing both cleaved and full forms of ATF6), and anti-XBP1 antibody (recognizing both non-spliced and spliced isoforms of XBP1). Immunohistochemistry assay was used to detect the expression changes of phospho-IRE1 (Fig. 2a), XBP1 (Fig. 2b), and ATF6 (Fig. 2c) in the cerebrum, cerebellum, and mesocephalon. Through positive pixel count and statistical analysis, we found that the protein level of phospho-IRE1 was higher in the cerebellum and mesocephalon of ZIKV-infected compared with the mock-infected (Fig. 2d). The protein level of XBP1 was higher in the cerebellum and mesocephalon of ZIKV-infected compared with the mock-infected (Fig. 2e). The protein level of ATF6 was higher in the mesocephalon of ZIKV-infected compared with the mock-infected (Fig. 2f). The results indicated that the expression of phospho-IRE1 and XBP1 significantly increased in the cerebellum and mesocephalon during ZIKV infection. The expression of ATF6 significantly increased in the mesocephalon. Activated ATF6n and XBP1s are translocated into the cell nucleus and regulated the transcription of target genes [32, 42]. In order to verify the activation of ATF6n and XBP1s, immunohistofluorescence was used to analyze the nuclear localization of XBP1s (Fig. 3a) and ATF6n (Fig. 3b) during ZIKV infection. The whole brain sections of AG6 mice were stained with DAPI (blue), anti-ZIKV envelope protein antibody (red), and anti-ATF6 or anti-XBP1 antibody (green). Images of the whole brain section were captured by using a digital slice scanning analysis system. We found that ZIKV infection stimulated ATF6n and XBP1s to translocate into the cell nucleus (Fig. 3c). These results further demonstrated that IRE1-XBP1 and ATF6 pathways were activated in the nervous tissues of the mouse brain during ZIKV infection, while ATF6n and XBP1s were translocated into the cell nucleus to target the downstream genes of UPR.

### ZIKV infection activates ER stress sensors in human neural cells

In order to verify the ER stress at the cellular level which occurred on the nervous tissues of the mouse brain during ZIKV infection, we selected two representative neural cells, neuroblastoma cell line (SK-N-SH), and astrocytoma cell line (CCF-STTG1), to perform experiments. Neural cells were infected with ZIKV at an MOI of 5 PFU/cell. Viral growth was analyzed at different hours post infection (hpi) via RT-qPCR for the quantification of the viral nucleic acid (see Additional file 2: a, b). The maximum virus RNA copies of SK-N-SH and CCF-STTG1 were 1.103 × 10^8^ copies/μg RNA at 48 hpi and 1.201 × 10^8^ copies/μg RNA at 36 hpi respectively. Confocal immunofluorescence microscopy also revealed the presence of envelope protein at 24 hpi (see Additional file 2: c, d). The results indicated that ZIKV could infect and replicate in SK-N-SH and CCF-STTG1 cells. These results offered a model to study the ER stress of ZIKV at the cellular level.

**Fig. 4:**
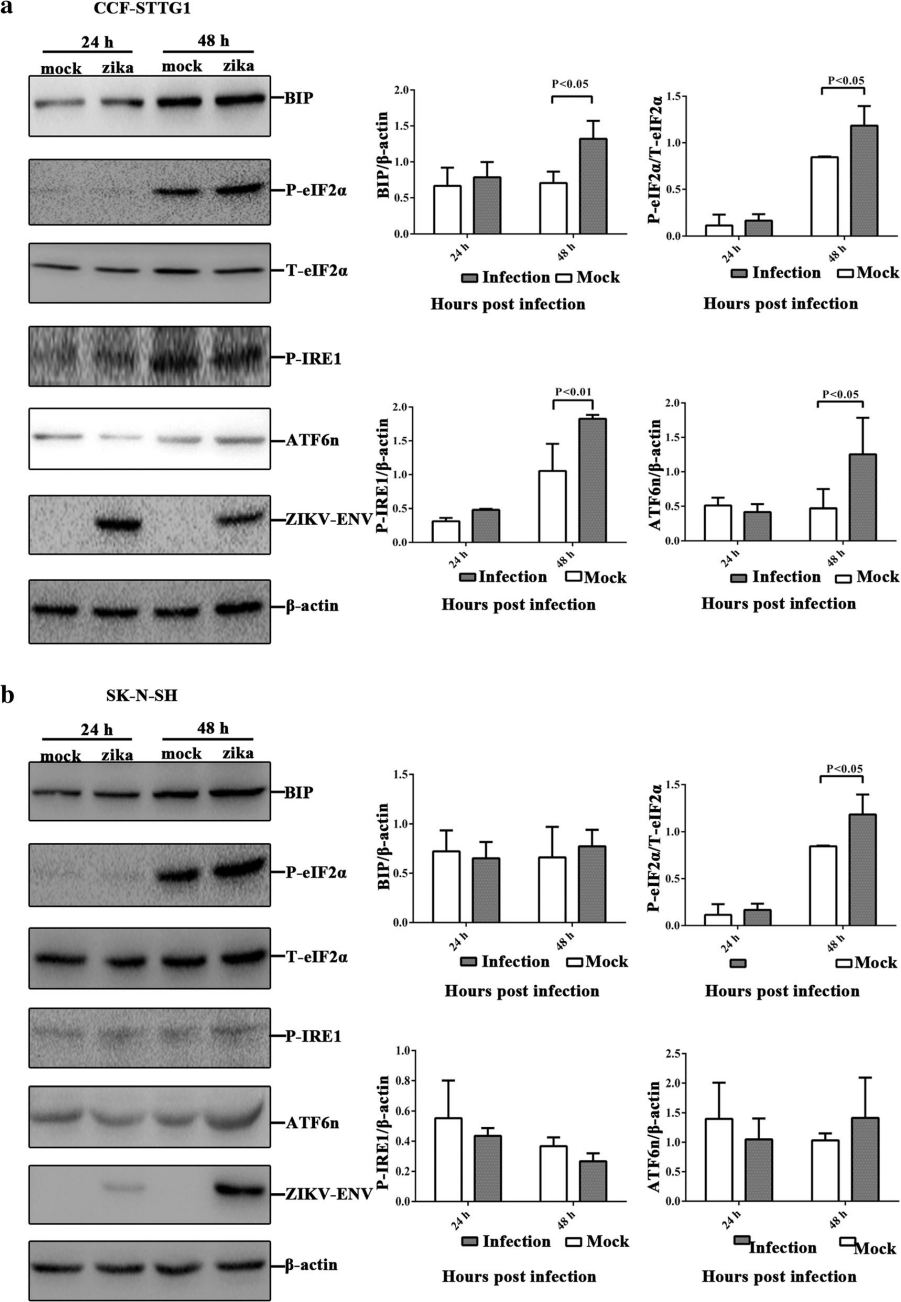
ZIKV infection increases the expression of ER stress sensors in human neural cells. **a**, **b** CCF-STTG1and SK-N-SH were infected with ZIKV at an MOI of 5 PFU/cell, and cellular lysates were obtained at 24 and 48 hpi. An equal amount of cell lysates were analyzed with anti-BIP antibody, anti-phospho-eIF2α antibody, anti-total-eIF2α, anti-phoshpo-IRE1 antibody, anti-ATF6n antibody, anti-ZIKV envelope protein antibody, and anti-β-actin antibody. The bands were analyzed by using Image Lab 4.0.1. The fold change of *target protein*/*β-actin* was calculated. The protein level between mock-infected and ZIKV-infected compared with internal control β-actin. Data represented three independent experiments and *error bars* indicate mean ± SD. Statistical analyses were performed using multiple *t* tests (*N* = 3) (*P* < 0.05 or *P* < 0.01)

To determine whether ZIKV infection in human neural cells also activates cellular ER stress, we detected the expression of ER stress sensors. Comparison of ZIKV infection with mock infection showed that the expression of BIP, phospho-elF2α, phospho-IRE1, and ATF6n significantly increased at 48 hpi in CCF-STTG1 (Fig. 4a). However, only phospho-elF2α significantly increased at 48 hpi in SK-N-SH (Fig 4b). The expression change of BIP, phospho-IRE1, and ATF6n was not significant in SK-N-SH. The results indicated that ZIKV infection strongly activated the UPR in CCF-STTG1 and slightly activated in SK-N-SH.

### ZIKV infection induces the splicing of *xbp1* and the translocation of XBP1s into the nuclei of human neural cells

**Fig. 5:**
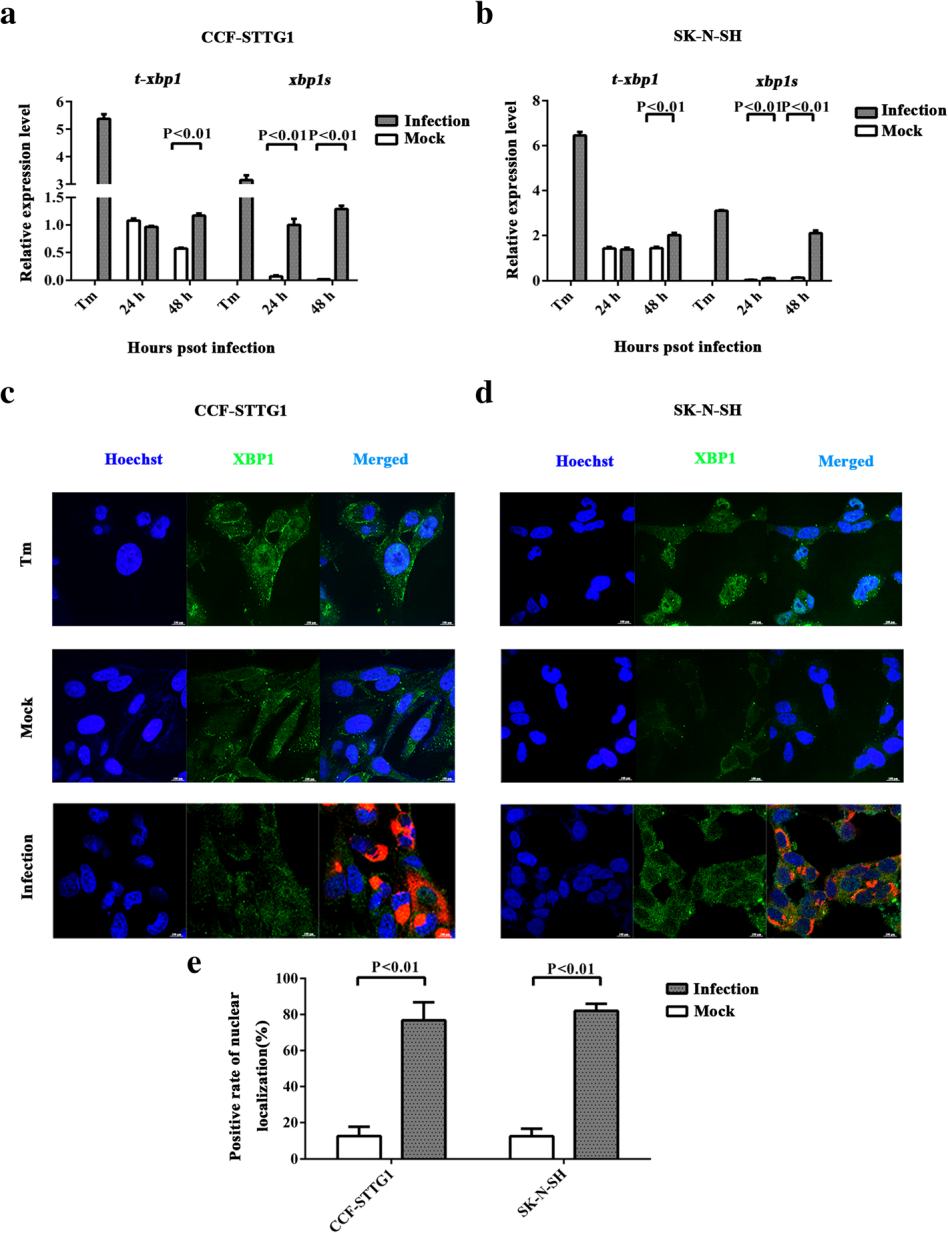
ZIKV infection induces the splicing of xbp1 and the translocation of XBP1s into the nuclei of human neural cells. **a**, **b** CCF- STTG1 and SK-N-SH were infected with ZIKV at an MOI of 5 PFU/cell. Positive control (Tm) was treated with 2 μM tunicamycin. RNA was extracted at 24 and 48 hpi and the first strand cDNA was synthesized. Primers that specifically amplified *xbp1s* and total *xbp1* (including both *xbp1u* and *xbp1s*) were used to analyze *xbp1s* via RT-qPCR. The relative expression levels of the *xbp1s* and *t-xbp1* were calculated according to the 2^−∆∆*Ct*^ method. The ratios of *xbp1s*/*t-xbp1* between mock-infected and ZIKV-infected were calculated. Data represented three independent experiments and *error bars* indicate mean ± SD. Statistical analyses were performed using multiple *t* tests (*N* = 3) (*P* < 0.05 or *P* < 0.01). **c**, **d** CCF- STTG1 and SK-N-SH were infected with ZIKV at an MOI of 5 PFU/cell and fixed at 24 hpi at which time *xbp1* starts to be spliced. Cells were stained with Goat anti-Rabbit IgG secondary antibody, FITC (green) against anti-XBP1 antibody (recognizing both non-spliced and spliced isoforms of XBP1) and Goat anti-Mouse IgG secondary antibody, Texas Red®-X (red) against anti-ZIKV envelope protein antibody. Cell nucleus was visualized using Hoechst 33258 (blue). Positive control (Tm) was treated with 2 μM tunicamycin. The results represent three independent experiments and one of three experiments is shown. **e** Positive rates of nuclear localization were counted. Data represent three independent experiments and *error bars* indicate mean ± SD. Statistical analyses were performed using multiple *t* tests (*N* = 3) (*P* < 0.05 or *P* < 0.01)

To determine whether the IRE-XBP1 pathway is activated in response to ZIKV infection in human neural cells, *xbp1s* was detected via RT-PCR. Similar to that induced by tunicamycin, the spliced *xbp1s* was detected at 24 and 48 hpi. The ratios of *xbp1s*/t-*xbp1* between mock and ZIKV infection were 15.8-fold (24 hpi) and 32.6-fold (48 hpi) in CCF-STTG1 cells, and 3.1-fold (24 hpi) and 10.9-fold (48 hpi) in SK-N-SH cells (Fig. 5a,b). RT-qPCR results indicated that *xbp1s* significantly increased during ZIKV infection. It implied that ZIKV infection induced the splicing of *xbp1*.

Confocal immunofluorescence microscopy was performed to further confirm the synthesis and nuclear localization of XBP1s. Guided by RT-qPCR data, SK-N-SH and CCF-STTG1 cells were grown on glass slides and infected with ZIKV at an MOI of 5 PFU/cell. The cells were fixed at 24 hpi, at which time *xbp1* starts to be spliced and stained by using Hoechst 33258 (blue), anti-ZIKV envelope protein antibody (red), and anti-XBP1 antibody (green) (recognizing both non-spliced and spliced isoforms of XBP1). XBP1 was located in the cytoplasm in the mock-infected group. However, XBP1s translocated into the cell nucleus in both ZIKV-infected CCF-STTG1 and SK-N-SH cells and positive controls (tunicamycin treatment) (Fig. 5c,d). Confocal immunofluorescence results showed that ZIKV infection induced the translocation of XBP1s in the cell nucleus, consistent with the positive controls. In contrast to ZIKV infection, mock infection did not induce the translocation of XBP1s in the cell nucleus (Fig. 5e). The results indicated that ZIKV infection-induced ER stress triggers the synthesis and transport of XBP1s into the cell nucleus.

### ZIKV infection activates the UPR downstream genes in the nervous tissues of the mouse brain and human neural cells

**Fig. 6:**
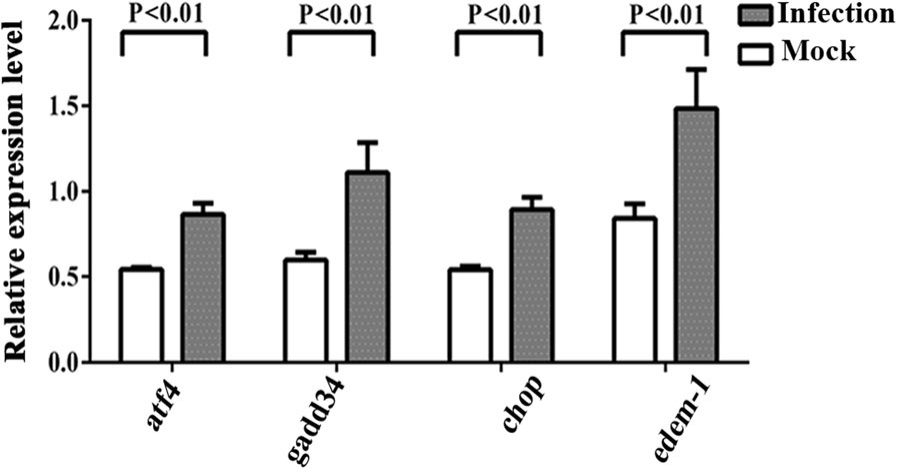
ZIKV infection activates UPR downstream genes in the mouse brain. Three-week-old AG6 mice were infected with 1 × 10^5^ PFU/mouse (each dose, *N* = 3) via intraperitoneal injection. The total RNA was extracted from the nervous tissues of the mouse brains. Following the synthesis of the first strand cDNA, RT-qPCR assay was performed to assess the transcriptional levels of downstream genes of ER stress, namely, *atf4*, *gadd34*, *chop*, and *edem-1*. The relative expression levels of genes were calculated according to the 2^−∆∆*Ct*^ method. Data represent three independent experiments and *error bars* indicate mean ± SD. Statistical analyses were performed using multiple *t* tests (*N* = 3) (*P* < 0.05 or *P* < 0.01)

**Fig. 7:**
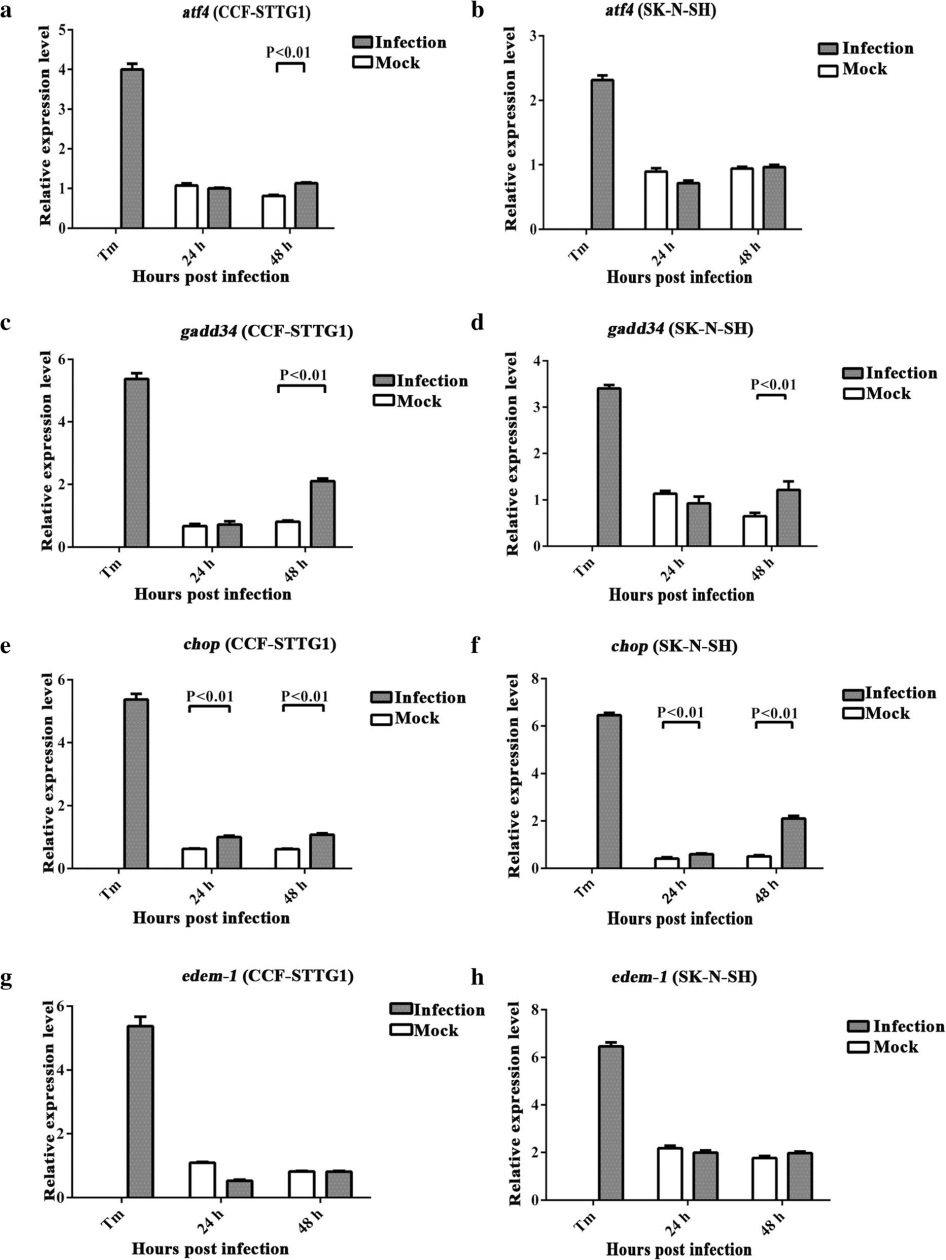
ZIKV infection activates the UPR downstream genes in human neural cells. **a**–**h** CCF-STTG1 and SK-N-SH were infected with ZIKV at an MOI of 5 PFU/cell. RNA was extracted at 24 and 48 hpi and the first strand cDNA was synthesized. Primers that specifically amplified *atf4* (**a**, **b**), *gadd34* (**c**, **d**), *chop* (**e**, **f**), and *edem-1* (**g**, **h**) were used. The relative expression levels of the genes were calculated according to the 2^−∆∆*Ct*^ method. Data represented three independent experiments and *error bars* indicate mean ± SD. Statistical analyses were performed using multiple *t* tests (*N* = 3) (*P* < 0.05 or *P* < 0.01)

Phosphorylated PERK mediates eIF2α phosphorylation and regulates many ER stress-related genes through ATF4, such as amino acid metabolism, *gadd34*, and cell apoptosis [43]. Our results indicated that ZIKV infection induced eIF2α phosphorylation in human neural cells which is similar with the previous study [44]. ATF4 is regulated by phospho-eIF2α, and its activation results in the function of the PERK-eIF2α pathway [16, 43]. To determine whether the increasing amount of phospho-beIF2α in ZIKV infection is sufficient to induce ATF4 translation, the mRNA level of *atf4* was measured using RT-qPCR. The results indicated that *atf4* significantly increased in the nervous tissues of the mouse brain (1.60-fold) (Fig. 6) and at 48 hpi in CCF-STTG1 cells (1.40-fold) but not in SK-N-SH cells (Fig. 7a,b,). Although the mRNA level of *atf4* increased slightly, the fold change was negligible. This result suggests that phospho-eIF2α weakly activates ATF4. GADD34 is a negative feedback loop that restores protein translation by targeting PP1 to phosphor-eIF2α and making it dephosphorylate [45, 46]. RT-qPCR results demonstrated that the mRNA level of *gadd34* significantly increased in the nervous tissues of the mouse brain (1.86-fold) (Fig. 6) and at 48 hpi (2.6-fold) in CCF-STTG1 (Fig. 7c) cells and at 48 hpi (1.9-fold) in SK-N-SH cells (Fig. 7d). Those results suggest that GADD34 expression is increased to restore protein translation during ZIKV replication, implying that ZIKV may take advantage of the protein synthesis of the host cell.

To deal with ER stress, the activated UPR causes translation attenuation, cellular apoptosis, and protein degradation. CHOP is one of the components of the ER stress-mediated apoptotic pathway. Activated CHOP further induces apoptotic markers, such as Bcl-2, Caspase-9, and Caspase-3 [16]. Its expression is mainly regulated at the transcriptional level. The transcriptional level of *chop* increased significantly in the nervous tissues of the mouse brain (1.65-fold) (Fig. 6) and at 24 hpi (1.6-fold) and 48 hpi (1.8-fold) in CCF-STTG1 cells (Fig. 7e), and at 24 hpi (1.5-fold) and 48 hpi (4.2-fold) in SK-N-SH cells (Fig. 7f). The transcription of *chop* was regulated by both ATF4 and XBP1s [16, 47]. However, ATF4 was weakly activated during ZIKV infection. The changing trends of *chop* were consistent with *xbp1s*. Thus, activated *chop* may be mainly regulated by XBP1s. ER degradation-enhancing α-mannosidase-like protein (EDEM) family proteins are the main members of ERAD activated during ER stress. XBP1 is involved in ERAD by the induction of EDEM [48]. Many studies demonstrated that EDEM-1 increases during virus infection or directly interacts with the viral proteins [49]. The transcriptional level of *edem-1* significantly increased in the nervous tissues of the mouse brain (1.76-fold) (Fig. 6), but no change was detected in neural cells (Fig. 7g,h). Therefore, the results implied that ZIKV infection mediates XBP1s to activate CHOP and may mediate cell apoptosis, but not EDEM-1 to activate protein degradation.

## Discussion

ZIKV is a mosquito-borne flavivirus which causes microcephaly in newborns and serious neurological complications in adults, such as Guillain–Barre syndrome. Although the relationship between ZIKV infection and microcephaly is demonstrated in vivo [50], the neuropathogenesis caused by ZIKV infection is still unclear. Ivan Gladwyn-Ng et al. reported that ZIKV induced ER stress and UPR in human cortices in vivo and in hNSCs in vitro, and their findings were confirmed in the mouse embryonic brain in vivo. They also investigated the mechanism of ZIKV-associated microcephaly by administration of pharmacological inhibitors of the UPR and found that the PERK-eIF2α pathway is the principal dependence [51]. In the current study, however, we observed that ZIKV infection activated the IRE1-XBP1 and ATF6 pathways in the mouse nervous tissues in vivo and human neural cell line in vitro. Those two pathways are different from the PERK-eIF2α for regulating ER stress, so we speculated that the UPR also may deal with ER stress in another way during ZIKV infection. Furthermore, it is reported that ER stress can directly initiate inflammatory pathways. In turn, pro-inflammatory stimuli can trigger ER stress and the resulting UPR activation can further amplify inflammatory responses [52]. However, because of the limitation of IFNR-deficient mice, we cannot investigate the interplay between the UPR activation and inflammatory responses, especially innate immune response and its role in ZIKV pathogenesis. In order to build a cellular model for the verification of ER stress in vitro, we demonstrated that ZIKV replication was highly efficient in human neural cells, SK-N-SH, and CCF-STTG1 cells. It is easy to study the neuropathogenesis caused by ZIKV infection in vitro by using this cellular model which is firstly reported. Using this cellular model, we investigated the activation and regulation of three UPR pathways in neural cells. We found that ZIKV infection in neural cells activated the IRE1-XBP1, PERK-eIF2α, and ATF6 pathways respectively. Comparing the effects of each pathway, we found that phospho-eIF2α-mediated transcriptional attenuation was slightly activated. IRE1-XBP1 and ATF6 pathways are mainly activated during ZIKV infection in vitro and in vivo. Considering their effect on cell apoptosis, protein degradation, and ER homeostasis mediated by IRE1-XBP1 and ATF6, we found *chop* was upregulated in vitro and in vivo. *Edem-1* was only upregulated in vivo. The chaperone, BIP, which participates in the regulation of ER homeostasis was upregulated in vitro and in vivo*.* Those results demonstrated that ZIKV infection activated the ER stress and regulated the UPR in neural cells.

### ZIKV infection activates the IRE1-XBP1 pathway to respond to ER stress

The IRE1-XBP1 signaling pathway plays an important role in viral infection. A number of viral infections activate IRE1-XBP1, triggering many cell responses. Firstly, ERAD activation accelerates protein degradation [53]. Secondly, CHOP expression induces apoptosis markers to guide cell death and plays a role in autophagy induction. Thirdly, IRE1 mediates the selective degradation of a subset of ER-located mRNAs. However, each virus causes different cell responses mediated by IRE1-XBP1. HCV glycoprotein E2 is an ERAD substrate that interacts with EDEM to accelerate glycoprotein degradation, which interferes with viral replication and particle production [53]. DENV infection induces CHOP but does not trigger apoptosis markers [16]. JEV induces the activation of the RIDD cleavage pathway, which is beneficial for viral infectivity [27, 49]. Findings in this study indicated that ZIKV infection induced the IRE1-XBP1 pathway to respond to cell stress. And the expression of ZIKV-ENV do not correlate with the XBP1 expression or its translocation into the nucleus, this suggested that ZIKV-ENV may not be responsible for induction of IRE1-XBP1 pathway or IRE1-XBP1 pathway activated in the infection state with the low expression level of ZIKV-ENV. The IRE1-XBP1 pathway triggered the cell responses of CHOP, which may activate cellular apoptosis. However, ZIKV infection did not trigger the EDEM-1 to increase the degradation of misfolded and unfolded proteins which alleviated the accumulation of abnormal proteins. We speculate that ZIKV infection facilitates neural cell apoptosis which may relate to the nerve injury and may also benefit from the virus release and contributes to viral replication [18].

### ZIKV infection activates the ATF6 pathway to respond to cell stress

The activation of ATF6 is critical to the UPR. ATF6 increases the expression of many ER chaperone genes. BIP, as a major ER chaperone, facilitates protein folding, preventing intermediates from aggregating, and promoting misfolded protein for proteasome degradation. BIP acts as a central regulator of ER homeostasis. BIP is essential for the correct protein folding of nascent viral peptides in the ER, including viral core protein expression. BIP also plays a role in the replication of viral genetic material as well as the formation of new viral capsid complexes [54]. Our results reveal that ATF6 and BIP upregulated during ZIKV infection in vitro and in vivo. ATF6 may promote the expression of BIP. Activated BIP expression sustains the ER homeostasis and contributes to the ZIKV replication. In addition, inducing the mRNA expression of *xbp1s* is one of the important functions of ATF6 [39, 49]. The mRNA level of *xbp1s* significantly increases, and XBP1s is translocated into the nucleus. ATF6n may participate in inducing *xbp1s* expression and mediate XBP1s activation.

### ZIKV infection induces the phosphorylation of eIF2α but does not increase the expression of ATF4

Phospho-eIF2α transiently attenuates global mRNA translation, thereby helping cells reduce the accumulation of misfolded proteins and cope with temporary ER stress [49]. DENV-2 infection suppresses PERK-mediated eIF2α phosphorylation [16]. We detected the phosphorylation of eIF2α and found that the level of phospho-eIF2α significantly increased during ZIKV infection. The mRNA level of *gadd34*, which recruits PP1 to dephosphorylate phospho-eIF2α, increased significantly during ZIKV infection. However, ATF4 was hardly activated during ZIKV infection. Therefore, eIF2α-ATF4 did not mediate translation attenuation. Inversely, ZIKV may suppress the translation attenuation through increasing the expression of GADD34.

Many neurological diseases are linked to ER stress. For example, the accumulation of misfolded protein such as β-amyloid, α-synuclein, and huntingtin is apparently associated with selective nerve cell death in Alzheimer’s, Parkinson’s, and Huntington’s diseases [47]. ATF6, XBP1, and CHOP play functional roles in controlling dopaminergic neuron survival [33]. In Parkinson’s disease studies, the activation of UPR maintains the protein homeostasis in ATF6-deficient mice. ATF6 controls the level of BIP and ERAD components in dopaminergic neurons [33]. We found that ZIKV infection activated the UPR in the mouse nervous tissues and verified in the neural cells. The results offer some clues to further study the mechanism of neuropathogenesis caused by ZIKV infection.

## Conclusions

Taken together, ZIKV infection significantly upregulated the expression of ER stress markers in the mouse nervous tissues and the neural cells. The results of this study provide evidence that ZIKV infection activated the UPR and offer some clues to further study the mechanism of neuropathogenesis caused by ZIKV infection.

## Additional files

Additional file 1: Immunohistochemistry assay for ZIKV infection in the mouse brain. a Three-week-old AG6 mice were infected with 1 × 10^5^ PFU/mouse (each dose, *N* = 3) via intraperitoneal injection. Sections of the cerebrum, cerebellum, and mesocephalon were obtained at 5 dpi, and anti-ZIKV envelope protein antibody was used for immunohistochemistry assay. Purple dots represent the cell nucleus. Brown spots represent the ZIKV envelope protein. Shown is representative one of three independent experiments. b Aperio ImageScope viewing software was used to analyze the percentage of positivity (algorithm, positive pixel count). Data represented three independent experiments and *error bars* indicate mean ± SD. Statistical analyses were performed using multiple *t* tests (*N* = 3) (*P* < 0.05 or *P* < 0.01). (EPS 474 kb)

Additional file 2: The model of ZIKV infection in human neural cells, CCF-STTG1, and SK-N-SH cell lines. a, b CCF-STTG1 and SK-N-SH were infected with ZIKV at an MOI of 5 PFU/cell. RNA was extracted and first strand cDNA was synthesized. A standard curve of RT-qPCR was built and the absolute quantification of the viral nucleic acid was calculated respectively. Data represented three independent experiments and *error bars* indicate mean ± SD. c, d Detection of viral envelope protein at 24 hpi by confocal immunofluorescence microscopy. The nucleus was visualized using Hoechst 33258 (blue), and the envelope protein was stained with the anti-ZIKV envelope protein antibody followed by Goat anti-Mouse IgG secondary antibody, Texas Red®-X (red). All the images were captured at × 10 magnification. One out of three independent experiments is shown, (c) CCF-STTG1 and (d) SK-N-SH. (EPS 1006 kb)

## Abbreviations

ATF4: Activating transcription factor 4
ATF6: Transcription factor 6
ATF6n: Cleaved ATF6
BIP: ER chaperone immunoglobulin heavy -chain-binding protein
BSA: Bovine serum albumin
CCF-STTG1: Astrocytoma cell line
CHOP: Pro-apoptotic transcription factor CCAT/enhancer-binding protein
DENV: Dengue virus
dpi: Days post infection
EDEM: ER degradation-enhancing α-mannosidase-like protein
eIF2B: Guanine nucleotide exchange factor
eIF2α: α -subunit of eukaryotic translation initiation factor 2
ER: Endoplasmic reticulum
ERAD: ER-association degradation RIDD: regulated IRE1-dependent decay
ERSEs: ER stress response elements
GADD34: Growth arrest and DNA damage-inducible protein
HCV: Hepatitis C virus
hpi: Hours post infection
HRP: Horseradish peroxidase
IRE1: Inositol-requiring enzyme 1
JEV: Japanese encephalitis virus
MOI: Multiplicity of infection
NGS: Normal goat serum
PERK: PKR-like ER kinase
phospho-eIF2α: Phosphorylated eIF2α
phospho-IRE1: Phosphorylated IRE1
PMSF: Phenylmethanesulfonyl fluoride
PP1: Protein phosphatase 1
SK-N-SH: Neuroblastoma cell line
TCID50: 50% tissue culture infectious dose
t-*xbp1*: Total *xbp1*
UPR: Unfolded protein response
UPREs: UPR elements
WNV: West Nile virus
XBP1: X-box binding protein 1
XBP1s: Spliced XBP1
*xbp1s*: Spliced *xbp1*mRNA
*xbp1u*: Unspliced *xbp1*mRNA
YFV: Yellow fever virus
ZIKV: Zika virus
ZIKV-ENV: ZIKV envelope protein
